# How to enhance the novices’ learning in ultrasound-guided procedures utilizing handmade phantoms?

**DOI:** 10.1186/s12909-024-06458-z

**Published:** 2024-12-18

**Authors:** Sih‑Shiang Huang, Chih-Hsien Lin, Shao-Yung Lin, Chien-Tai Huang, Wan-Ching Lien

**Affiliations:** 1 Department of Emergency Medicine, National Taiwan University Hospital and National Taiwan University, No.7, Chung-Shan South Road, Taipei, 100 Taiwan; 2 Department of Emergency Medicine, National Taiwan University Hospital, Hsin-Chu Branch, Hsin-Chu City, Taiwan; 3 Department of Emergency Medicine, College of Medicine, National Taiwan University, Taipei, Taiwan

**Keywords:** Phantom, Ultrasound, Procedure, Thoracocentesis, Pericardiocentesis

## Abstract

**Introduction:**

This prospective study aims to evaluate the learning effect of US-guided thoracocentesis and pericardiocentesis in novices through simulation training using handmade phantoms.

**Methods:**

The novices included undergraduate-year (UGY) students and first postgraduate-year (PGY-1) residents. Handmade phantoms were utilized for training and immediate assessment. Novices were re-evaluated using high-fidelity phantoms three months after training, while experienced PGY-3 emergency medicine residents were recruited and evaluated with high-fidelity phantoms simultaneously. Data on their performance, puncture time, and number of attempts were collected.

**Results:**

Thirty-six novices (18 PGY-1 and 18 UGYs) and 12 PGY-3 emergency medicine residents were recruited. Alongside clinical observation, novices demonstrated improved skill retention and performance at the 3-month assessment compared to the immediate assessment [5 (4–5) vs. 3.5 (3–4), *p* = 0.0005] in thoracocentesis, achieving a comparable level of proficiency with the PGY-3 emergency medicine residents [5 (4–5) vs. 5 (5), *p* = 0.105]. Without clinical observation, novices exhibited a decline in skill proficiency in pericardiocentesis at the 3-month assessment [3 (3–4) vs. 4 (4–4.5), *p* = 0.015]. The puncture time was comparable between novices and PGY-3 emergency medicine residents for both thoracocentesis and pericardiocentesis. However, novices required a greater number of puncture attempts for pericardiocentesis.

**Conclusions:**

Novices showed superior performance in thoracocentesis but experienced skill decay in pericardiocentesis at the 3-month assessment following training with handmade phantoms. This decline may be attributed to the very low frequency of pericardiocentesis cases encountered by novices after training, as well as the higher-stakes nature of the procedure. Further investigation is needed to evaluate the long-term effects of training, skill retention, and transfer of skills to actual patient care. Additionally, research should focus on determining optimal retraining intervals for pericardiocentesis and evaluating the use of standardized pericardiocentesis videos as an alternative to clinical observation.

**Trial registration:**

Registered at ClinicalTrials.gov (NCT04792203) on March 7, 2021.

**Supplementary Information:**

The online version contains supplementary material available at 10.1186/s12909-024-06458-z.

## Introduction

Ultrasound (US)-guided clinical procedures play a crucial role in emergency medicine (EM) education [1]. Ensuring patient safety is of utmost importance, particularly when inexperienced physicians are directly involved in performing procedures. To establish a safe approach, novices should prioritize the development of hand–eye coordination and psychomotor skills before applying these procedures to real patients [2].

Simulation provides a secure environment for acquiring skills, facilitates the learning of complex procedures, and reduces anxiety, especially among vulnerable learners [3, 4]. It significantly contributes to developing proficiency in US-guided procedures [5]. However, commercially developed high-fidelity phantoms can be expensive, often exceeding USD 3,000, which poses challenges for many emergency departments (EDs) with budget constraints.

Handmade phantoms have been successfully developed for US-guided biopsy, thoracocentesis, and pericardiocentesis [5–16]. However, there has been limited research on the learning effects of handmade phantoms [3]. The performance of novices is a topic of interest, especially when compared to that of experienced sonographers. Furthermore, it remains unclear whether the learning effect varies across different procedures.

This study aims to evaluate the performance of novices after training with handmade phantoms for US-guided thoracocentesis and pericardiocentesis. The performance was assessed using a high-fidelity phantoms 3 months later and compared with that of experienced residents.

## Methods

This prospective study was conducted at the ED of the National Taiwan University Hospital (NTUH) from August 2022 to July 2023. It was approved by the institutional review board of the NTUH (202011111RIND) and registered at ClinicalTrials.gov (NCT04792203). Informed consent was obtained from each participant. US machines (Xario 100, Canon, Japan, and Arietta 780, Fujifilm Healthcare, Japan) equipped with a 2–5 MHz curvilinear transducer were used.

### Agar substrate preparation

The agar substrate was prepared using 1% w/v agar powder (Fairsen Foods Industry Co., Ltd) by dissolving 10 g of agar powder in 1000 mL of water. After thoroughly heating to dissolve the agar powder and subsequent filtration to remove impurities, the resulting clear solution was tinted with 2 teaspoons of dark blue food coloring (Ever Style Foodstuff Industrial Co., Ltd).

### Thoracocentesis phantom construction

**Fig. 1:**
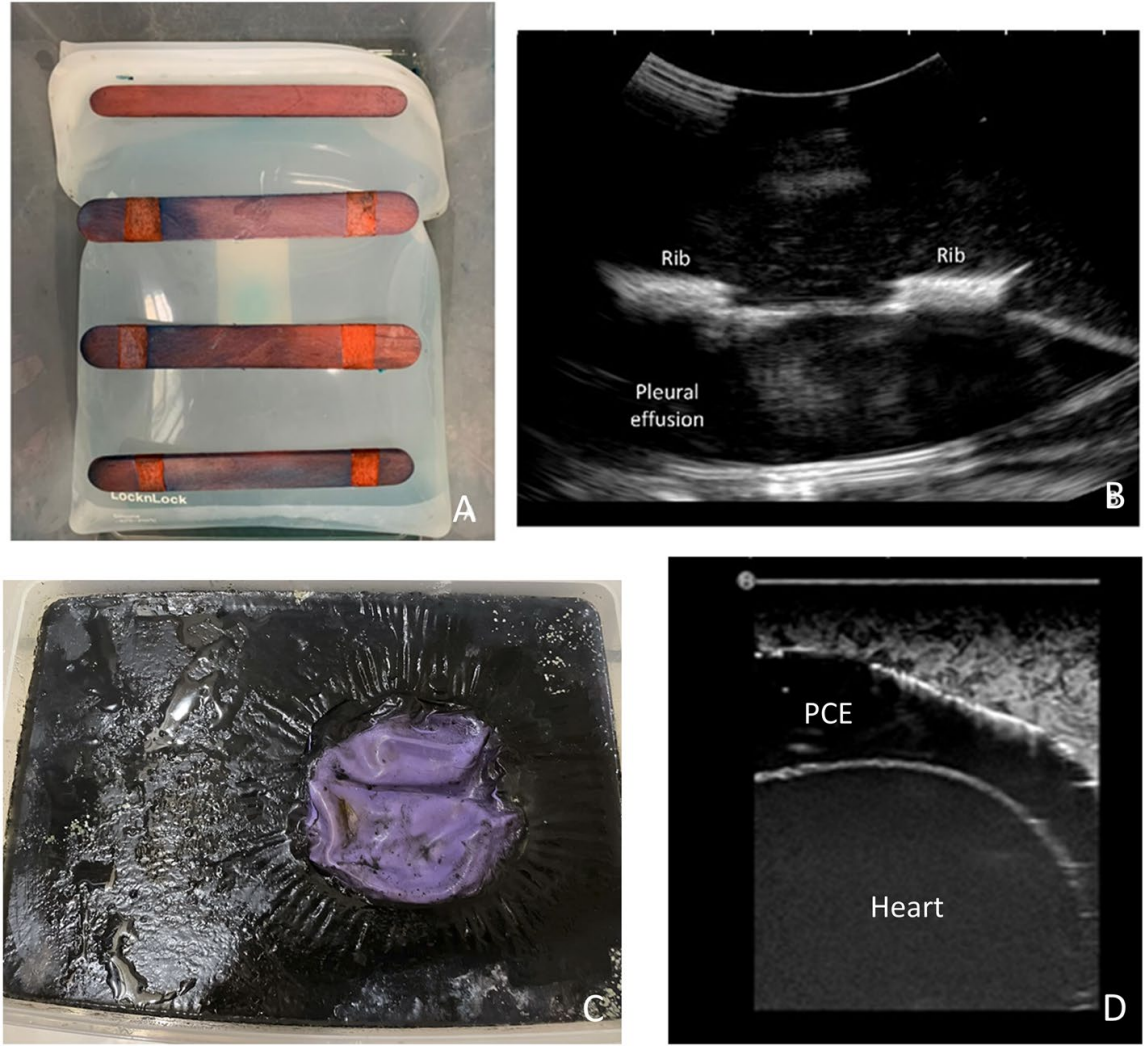
**A** The appearance of the thoracocentesis phantom with the silicone Ziploc bag and tongue depressors; **B**, The sonographic image of the thoracocentesis phantom; **C**, The appearance of the pericardiocentesis phantom with the outer balloon; **D**, The sonographic image of the pericardiocentesis phantom

A 15 × 10 × 10 cm silicone Ziploc bag filled with water tinted yellow using 2 teaspoons of food coloring (Ever Style Foodstuff Industrial Co., Ltd) was used to simulate pleural effusion. Tongue depressors, spaced at 1 cm intervals, were attached to the bag using super glue (FLEX SEAL®) to mimic ribs. The Ziploc bag was securely fixed at the bottom of a 35 × 25 × 15 cm container with super glue (Fig. 1A). The container was then filled with the prepared agar substrate, allowing for adjustment of thickness based on different body habitus. After assembly, the phantom was refrigerated for 4 h to increase longevity before use. The US images of the phantoms were reviewed by experts prior to use to ensure they resembled those of real patients (Fig. 1B).

### Pericardiocentesis phantom construction

Drawing inspiration from existing literature [7, 17], this phantom utilized a 3-inch balloon filled with red-dyed water using 2 teaspoons of food coloring (Ever Style Foodstuff Industrial Co., Ltd) to simulate blood in the heart. The small balloon was placed within a 6-inch balloon filled with yellow-dyed water using 2 teaspoons of food coloring (Ever Style Foodstuff Industrial Co., Ltd), representing pericardial effusion. The larger balloon was securely attached to the bottom of a 35 × 25 × 15 cm container with super glue (FLEX SEAL®), and agar substrate was added to cover the balloon (Fig. 1C). After refrigerating for 4 h, the phantom was ready for use. The US images of the phantoms were reviewed by experts prior to use to ensure they resembled those of real patients (Fig. 1D). Supplementary Table 1 provides detailed cost information for the phantoms.

### Novices recruitment and simulation curriculum for US-guided procedures

The novices, recruited from NTUH through public billboards, willingly participated in the study. They included undergraduate-year (UGY) students (i.e. the sixth-year medical students) and first postgraduate-year (PGY-1) residents with experience in performing fewer than 20 US scans. None had prior experience with US-guided procedures on phantoms or real patients. The PGY-1 participants came from various specialties and were not limited to emergency medicine.

Before the curriculum for US-guided procedures, novices completed a survey assessing self-reported confidence levels on a 5-point Likert scale (1 = unable to perform the procedure or lacks confidence entirely; 2 = performs the procedure with significant difficulty and requires considerable assistance; 3 = performs the procedure with moderate difficulty and occasional assistance; 4 = performs the procedure competently with minimal guidance; 5 = performs the procedure independently and confidently with high proficiency). Subsequently, a small-group hands-on training session (instructor-to-novice ratio of 1:4) utilizing the hand-made phantoms was conducted following a 30-min didactic session.

After the curriculum, all novices underwent a skill test session, performing thoracocentesis and pericardiocentesis on hand-made phantoms. Performance was evaluated using assessment forms developed based on a literature review [17] and expert consensus (Supplementary Tables 2 and 3). Two instructors, not involved in the enrollment and training, independently assessed the performance, with one on-site and the other evaluating videos with novices' faces masked. Puncture time, from the initiation of the attempt to fluid aspiration, and the number of puncture attempts were recorded. Novices reported their confidence levels and provided feedback on the phantom and curriculum after the curriculum, using a 5-point Likert scale (1 = strongly disagree; 2 = disagree; 3 = neutral; 4 = agree; 5 = strongly agree). Additionally, novices received feedback on their performance after the curriculum.

Three months after completing the curriculum, the novices’ performance was re-evaluated using a commercial high-fidelity phantom (MW-17, Kyoto Kagaku, Japan). Their experiences with US-guided thoracocentesis and pericardiocentesis during the 3 months following training were documented. Concurrently, 12 experienced EM residents (the third PGY, PGY-3) were recruited without any handmade phantom training. Their prior experience in US-guided procedures was collected. Their performance using the commercial phantom was assessed, and the puncture time and the number of puncture attempts were recorded. Novices and PGY-3 received feedback on their performance after 3-month assessments.

### Sample size estimation

To achieve 80% statistical power at a 5% significance level, a sample size of 8 per group was calculated based on an expected improvement in global ranking scores from a mean of 1.25 to 3.08, with an anticipated standard deviation of 1.24 after training with low-fidelity phantoms [3].

### Statistical analysis

All data were analyzed by SAS software (SAS 9.4, Cary, North Carolina, USA). Initially, we conducted the Shapiro–Wilk test to assess the normality of continuous data. If the data did not conform to a normal distribution, we presented it using medians and interquartile ranges (IQRs). Wilcoxon's rank-sum test was employed for comparing PGY-3 EM residents and novices, while the Wilcoxon signed-rank test was used for paired data.

Inter-rater reliability for both the items on the assessment form and global scores was evaluated using the intraclass correlation coefficient (ICC) with 95% confidence intervals (CIs). The Spearman correlation coefficient was used to evaluate the relationship between the total score and the global score. The total score represented the sum of each item on the assessment form. The internal reliability of the assessment form was estimated by employing Cronbach’s alpha coefficient [18]. A *p*-value less than 0.05 was considered statistically significant.

## Results

**Fig. 2:**
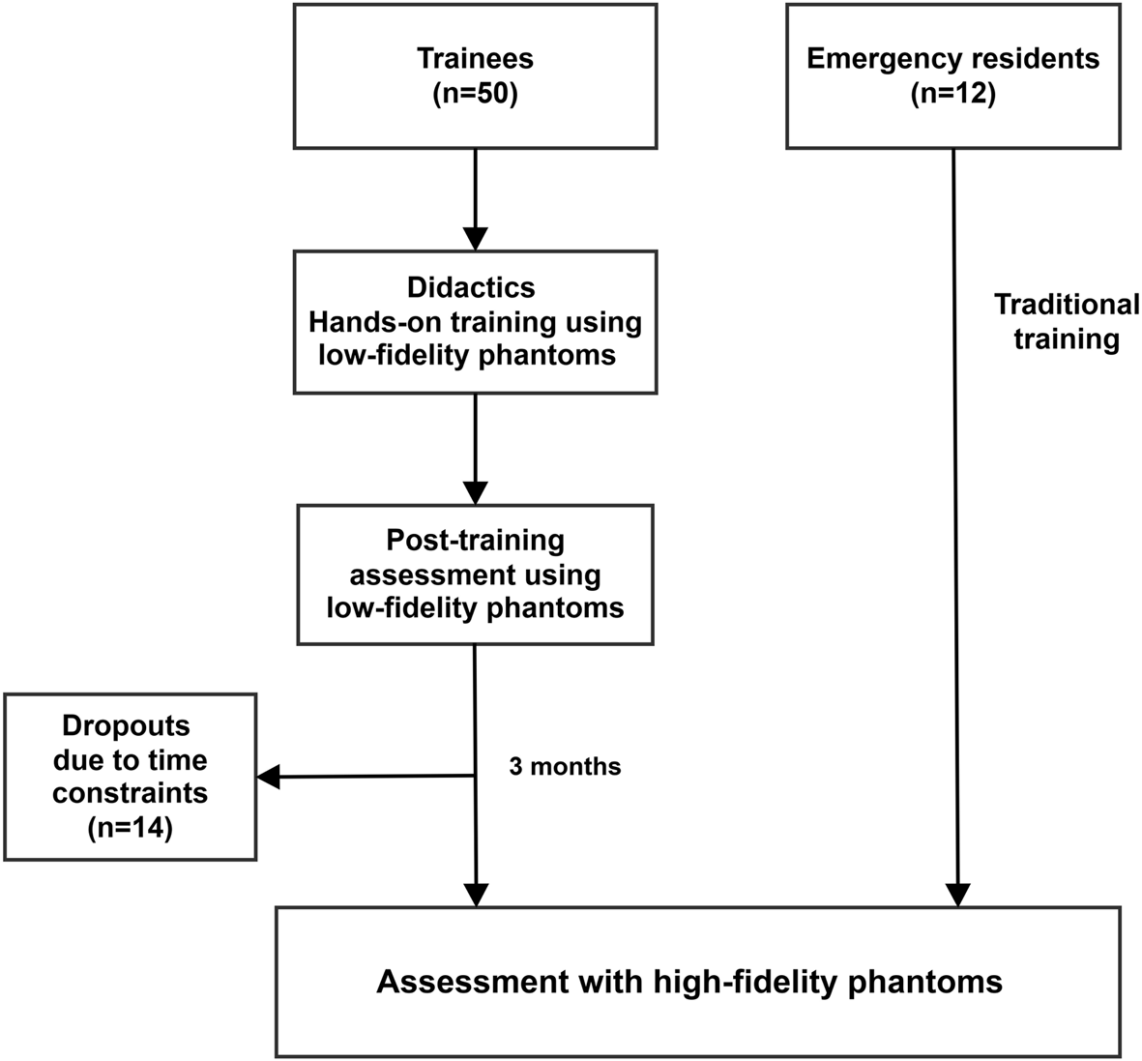
The study flowchart

Fifty novices, comprising 28 UGYs and 22 PGY-1 residents, were participated in the curriculum. Fourteen novices were unable to complete the 3-month assessment due to time constraints. The final analysis included 36 novices (18 UGY and 18 PGY-1) who completed both the immediate and 3-month assessments. Additionally, 12 clinically experienced, PGY-3 EM residents were recruited and evaluated (Fig. 2).

After the curriculum, the novices observed thoracocentesis a median of 2 times (ranging from 1 to 2 times) per month. However, they did not observe any pericardiocentesis. In contrast, the PGY-3 EM residents, who had prior experience with US-guided procedures on more than 20 real patients, performed US-guided thoracocentesis of a median of 8 times (ranging from 5 to 10 times) and pericardiocentesis 1 time (ranging from 1 to 2 times) per month.

The scores for each item on the assessment form, the global score, puncture time, the number of puncture attempts, and feedback were not normally distributed (all *p* < 0.0001 using the Shapiro–Wilk test). Therefore, these data were presented using medians and IQRs.

### Assessment forms

The ICCs for the each items of the assessment forms for thoracocentesis and pericardiocentesis were all more than 85%, indicating strong inter-rater reliability (Supplementary Tables 4 and 5). The Spearman correlation coefficients were 0.86 (95% CI, 0.75–0.92) and 0.90 (0.82–0.94) between the total score and the global score for thoracocentesis and pericardiocentesis, respectively, suggesting a high correlation. The standardized Cronbach’s alpha coefficient were 0.71 in thoracocentesis and 0.75 in pericardiocentesis, suggesting good internal reliability.

### Performance of the novices in thoracocentesis

The novices (PGY-1 and UGY) had significant improvements in visualization of pleura, lung, and effusion, puncture and fluid aspiration, needle steadiness during aspiration, global rating scale scores and less puncture time at the 3-month assessment. Also, they had comparable performance with experienced PGY-3 EM residents (Table 1). In a subgroup analysis, there was no significant difference in performance between novice PGYs and UGYs, both immediately after training and at the 3-month assessment (Table 2).

**Table 1:** The performance of the novices including the first postgraduate year (PGY-1) residents and undergraduate year (UGY) students, and PGY-3 emergency medicine residents in thoracocentesis

Variable	Novices (*n* = 36)	PGY^†^-3 EM^†^ residents (*n* = 12)	*p*-value
Immediate assessment using a low-fidelity phantom^*^			
Visualization of pleura, lung, and effusion	4 (3–4)^‡^		
Visualization of needle	5 (5)		
Puncture and fluid aspiration	4 (3–4.5)^§^		
Needle steadiness during aspiration	5 (4–5)^a^		
Total score	17 (16–18)^b^		
Global rating	3.5 (3–4)^c^	-	
Puncture attempt	1 (1–2)		
Puncture time (sec)	57 (41–122)^d^	-	
3-month assessment using a high-fidelity phantom^*^			
Visualization of pleura, lung, and effusion	5 (3–5)^‡^	5 (5)	0.164
Visualization of needle	5 (5)	5 (5)	0.382
Puncture and fluid aspiration	5 (4–5)^§^	5 (5)	0.562
Needle steadiness during aspiration	5 (5)^a^	5 (5)	0.136
Total score	20 (16–20)^b^	20 (20)	0.177
Global rating^*^	5 (4–5)^c^	5 (5)	0.105
Puncture attempt	1 (1)	1 (1)	1.000
Puncture time (sec)^*^	13 (9.5–30.5)^d^	14 (10–20)	0.232

^*^presented with median and interquartile ranges

^†^*PGY *Postgraduate year, *EM *Emergency medicine

^‡^*p* = 0.028

^§^*p* = 0.008

^a^*p* = 0.046

^b^*p* = 0.007

^c^*p* = 0.0005

^d^*p* < 0.0001

**Table 2:** The performance of the novice first postgraduate-year (PGY-1) residents and undergraduate year (UGY) students

Variables	PGY-1^†^ (*n* = 18)	UGY^†^ (*n* = 18)	*p*-value
Thoracocentesis			
Low-fidelity phantom			
Visualization of pleura, lung, and effusion	3 (3–5)	4(4)	0.577
Visualization of needle	5 (4–5)	5 (5)	0.270
Puncture and fluid aspiration	3 (3–4)	4 (3–5)	0.353
Needle steadiness during aspiration	4 (3–5)	5 (4–5)	0.173
Total score	16 (15–18)	18 (16–18)	0.208
Global rating^*^	3 (3–4)	4 (3.5–4)	0.095
Puncture attempt	1 (1–2)	1 (1–2)	1.000
Puncture time (sec)^*^	55 (43–115)	60 (39–150)	0.665
High-fidelity phantom			
Visualization of pleura, lung, and effusion	4 (3–5)	5 (3–5)	0.181
Visualization of needle	5 (3–5)	5 (5)	0.218
Puncture and fluid aspiration	5 (3–5)	5 (5)	0.710
Needle steadiness during aspiration	5 (5)	5 (5)	1
Total score	19 (14–20)	20 (18–20)	0.331
Global rating^*^	4.5 (4–5)	5 (4–5)	0.680
Puncture attempt	1 (1)	1 (1)	1.000
Puncture time (sec)^*^	13 (9–33)	13 (10–30)	0.721
Pericardiocentesis			
Low-fidelity phantom			
Visualization of heart, pericardium, and effusion	4 (3–5)	4 (4–5)	0.569
Visualization of needle	4 (4–5)	4 (4–4.5)	0.426
Puncture and fluid aspiration	4 (3–4)	4.5 (4–5)	0.107
Needle steadiness during aspiration	3 (3–3.5)	4 (3–5)	0.023
Total score	17 (13–17.5)	17 (15–19)	0.246
Global rating^*^	4 (3–4.5)	4.5 (4–5)	0.051
Puncture attempt	2 (1–2)	1 (1–2)	0.280
Puncture time (sec)^*^	113 (59–203)	67 (45–117)	0.033
High-fidelity phantom			
Visualization of heart, pericardium, and effusion	5 (3–5)	5 (3–5)	0.692
Visualization of needle	5 (5)	5 (3–5)	0.351
Puncture and fluid aspiration	4 (3–5)	4 (3–5)	1
Needle steadiness during aspiration	5 (3–5)	4 (3–5)	0.726
Total score	17 (14–20)	16 (14–20)	0.596
Global rating^*^	3 (2.5–5)	3 (3–4)	0.898
Puncture attempt	2 (1–2)	2 (1–2)	0.906
Puncture time (sec)^*^	62 (30–129)	47 (19–108)	0.389

^*^presented with median and interquartile ranges

^†^*PGY-1*  First postgraduate year, *UGY*  Undergraduate year

### Performance of the novices in pericardiocentesis

Novices (PGY-1 and UGY) had skill decay at the 3-month assessment in global scores. The PGY-3 EM residents demonstrated better performance in visualizing the heart, pericardium, and effusion, showed greater needle steadiness during aspiration, received higher global rating scores, and required fewer puncture attempts (Table 3). There was no significant difference in the performance between the PGYs and UGYs except for needle steadiness during aspiration and puncture time on the low-fidelity phantoms (Table 2).

**Table 3:** The performance of the novices including the first postgraduate year (PGY-1) residents and undergraduate year (UGY) students, and PGY-3 emergency medicine residents in pericardiocentesis

Variable	Novices (*n* = 36)	PGY^‡^-3 EM^‡^ residents (*n* = 12)	*p*-value
Immediate assessment using a low-fidelity phantom^*^			
Visualization of heart, pericardium, and effusion	4 (4–5)		
Visualization of needle	4 (4–5)		
Puncture and fluid aspiration	4 (4–5)		
Needle steadiness during aspiration	3.5 (3–4.5)		
Total score	17 (15–17.5)		
Global rating	4 (4–4.5)^†^	-	
Puncture attempt	2 (1–2)		
Puncture time (sec)	85 (45–144.5)	-	
3-month assessment using a high-fidelity phantom^*^			
Visualization of heart, pericardium, and effusion	5 (3–5)	5 (5)	0.038
Visualization of needle	5 (3–5)	5 (5)	0.504
Puncture and fluid aspiration	5 (3–5)	5 (5)	0.139
Needle steadiness during aspiration	5 (3–5)	5 (5)	0.016
Total score	16 (14–20)	20 (19–20)	0.031
Global rating	3 (3–4)^†^	5 (5)	0.0002
Puncture attempt	2 (1–2)	1 (1)	0.002
Puncture time (sec)	57 (28–117)	48 (31–103)	0.616

^*^presented with median and interquartile ranges

^†^*p* = 0.015

^‡^*PGY *Postgraduate year, *EM *Emergency medicine

### Feedback of the novices (PGY-1 and UGY)

The novices reported a significant increase in Likert-rated confidence in performing thoracocentesis/pericardiocentesis [before vs. after the curriculum, 1 (1) vs. 4 (4), *p* < 0.0001]. Also, the novices reported high satisfaction with the curriculum and phantom fidelity except for the puncture texture (Table 4).

**Table 4:** The feedback of the novice first postgraduate-year (PGY-1) residents and undergraduate year (UGY) students

Variables	Total (*n* = 36)	PGY^§^-1 (*n* = 18)	UGY^§^ (*n* = 18)	*p*-Value
Competency^*^				
Pre-curriculum confidence^†^	1 (1)	1 (1)	1 (1)	1.000
Post-curriculum confidence^†^	4 (4)	4 (4–5)	4 (3.5–4)	0.449
Curriculum^*^				
The curriculum is adequate for training	4 (4–5)	5 (4–5)	4 (4–5)	0.878
The curriculum would enhance coordination	4 (4–5)	4 (4–5)	4 (3.5–5)	0.978
The curriculum would enhance patient safety	4 (3–5)	4 (4–5)	4 (3–5)	0.667
The necessity of the training	5 (4–5)	5 (4–5)	5 (4–5)	0.805
Phantom fidelity^*^				
Image simulation mimicking human tissues	4 (3–4)	4 (3–4)	4 (3–4)	0.388
Puncture texture mimicking human tissues	3 (3–4)	4 (3–4)	3 (3–4)	0.353
Puncture needle visualization	4 (3–5)	4 (4–5)	4 (3–5)	0.601
Fluid drainage simulation	4 (3–4)	4 (3–5)	3 (3–4)	0.072
Endurance for repeat puncture	4 (3–5)	4 (3–5)	4 (3–5)	0.523

^*^presented with median and interquartile ranges

^†^*p* < 0.0001

^§^*PGY *Postgraduate year; *UGY*  Undergraduate year

## Discussion

This prospective study investigates the learning outcomes of novices in US-guided thoracocentesis and pericardiocentesis. The utilization of handmade phantoms was found to significantly enhance the learning process for US-guided procedures at the immediate assessment. However, a noticeable difference in learning effectiveness was observed between the two procedures. Novices displayed improved performance in thoracocentesis during the 3-month assessment compared to the immediate assessment. Furthermore, novices demonstrated performance comparable to that of experienced residents in thoracocentesis; however, their performance in pericardiocentesis did not reach a similar level and even declined. This variance may be attributed to the very lower frequency of pericardiocentesis cases they encounter, as well as its classification as a higher-stakes procedure. On the other hand, the observed differences in learning suggest that training for pericardiocentesis may need to be conducted more frequently, incorporate extended simulation sessions, or utilize standardized pericardiocentesis videos as an alternative to clinical observation to maintain proficiency and prevent skill decay.

Simulation-based learning for clinical procedures has demonstrated enhanced performance and has been linked to improved patient-level outcomes [4, 19, 20]. Through simulation learning, novices retain not only knowledge but also its practical application [3]. Novices learn the motor sequences necessary to manipulate the US transducer to achieve visual standards, thereby developing US muscle memory [19]. However, novices may experience cognitive overload from managing transducer movements, machine operation, and learning US anatomy simultaneously [21, 22]. Our simulation curriculum concentrated on two specific procedural tasks, offering a low-pressure and secure environment for novices. By utilizing handmade phantoms with highly realistic sonographic images, the confidence was boosted and anxiety was reduced.

Currently, commercial high-fidelity simulators remain prohibitively expensive for many training centers. Handmade phantoms serve as a cost-effective alternative for training purposes. The results indicated comparable puncture times in pericardiocentesis and thoracocentesis between novices and experienced residents, suggesting an improvement in novices' psychomotor skills when utilizing handmade phantoms.

Thoracentesis is a frequently performed procedure, with up to 173,000 cases annually in the United States [23]. Skills required for thoracocentesis involve maneuvering the transducer in response to the displayed image and the tactile sensation of a rib. In contrast, pericardiocentesis is a more advanced and challenging procedure. It requires identifying anatomical landmarks, distinguishing surrounding tissues—such as the diaphragm, liver, gastrointestinal tract, and lung—while avoiding direct puncture of the moving heart, and skillfully manipulating the transducer. Pericardiocentesis is a technical procedure included in the cardiology specialty [24]. A 10-year survey of emergency residents found an annual average of only 4 pericardiocentesis procedures, highlighting its rarity [25]. However, as our hospital is a tertiary medical center, EM residents here performed pericardiocentesis more frequently than the reported annual average.

In our study, novices demonstrated superior performance in thoracocentesis at the 3-month assessment, achieving comparable levels to the experienced residents. In contrast, their performance in pericardiocentesis declined and did not reach the experienced residents' level. Two possibilities could explain these differences. Firstly, pericardiocentesis is a rare procedure, and during the 3-month period, the novices included in our study did not have the opportunity to observe the procedure in the clinical setting. Direct observation of clinical procedure processing contributes to the maintenance and enhancement of psychomotor skills, as stated by the constructivist learning theory [26]. The incorporation of simulation-based training alongside clinical training results in sustained enhancements in performance [27]. Secondly, the phantoms used for pericardiocentesis in our study only simulated the heart and pericardial effusion, without including surrounding tissues, whereas high-fidelity phantoms did include these proximities. Wang et al. suggested that complex tasks might be better learned using higher-fidelity simulations, which offer greater cognitive stimuli [2]. Whether handmade phantoms are more suitable for low-stakes procedures (e.g., thoracocentesis) for novices remains a topic of debate [28]. Our findings suggest that low-fidelity phantoms can provide training outcomes comparable to those of high-fidelity phantoms for thoracocentesis. However, they may not be adequate for training in pericardiocentesis, a less frequently performed and more complex procedure.

Moreover, procedures are encompassed within the realm of patient care in emergency medicine milestones [29]. US-guided procedures are integral to the core application of emergency US [1]. However, comprehensive details regarding US-guided procedures are still lacking. Our findings offer evidence concerning milestone designation for US-guided procedures, suggesting that thoracocentesis could be classified at level 1, while pericardiocentesis would likely be categorized at level 2 or higher.

Furthermore, the puncture time and the global scores of the novices for pericardiocentesis were inconsistent, compared with those of experienced residents. This could be attributed to the lack of skill in transducer handling and acquiring clear images before the puncture, as well as the need for more puncture attempts.

Few studies have investigated skill retention using handmade phantoms [3]. Our results demonstrated that the skill for thoracocentesis was maintained after 3 months of the curriculum and showed a medium-term learning effect in both PGYs and UGYs. As a previous study reported that earlier procedural training would lead to more productive residents [30], our findings suggest that "the earlier" might begin as early as UGY, during the pre-clinical phase of physician development.

The novices in our study reported a significant increase in confidence, high satisfaction with the simulation curriculum, and phantom fidelity, except for puncture texture. Although our agar phantom had a semi-firm texture, the tactile sensation would not entirely simulate human skin. However, the needle could be visualized and tracked on the screen, aligning with the goals of US-guided procedure training. Commercial phantoms typically use polymers as ingredients, resulting in an overly firm texture. Covering the handmade phantoms with pig skin could be a potential solution, warranting further investigation.

There are limitations to this study. First, a randomized design would yield stronger conclusions. In this study, however, we did not randomize the novices due to budget constraints that limited our access to high-fidelity phantoms at the outset. Second, the novices were recruited from a single medical center and volunteered to join the study, potentially leading to selection bias and heightened motivation. However, this bias was mitigated by comparing them with experienced residents from the same institution who were also voluntarily recruited. Third, the comparison group consisted of EM residents. At our hospital, EM residents have the most extensive experience with US-guided procedures. The conclusions might have been more robust if the resident group had included individuals with a broader range of experience to account for variability within the group. Also, including participants from a broader range of medical training levels, such as residents and clinicians from various specialties, would enhance the generalizability of our findings. Fourth, while novices demonstrated statistically significant improvement in thoracocentesis performance at the 3-month assessment compared to the immediate assessment, caution is needed when interpreting these results and translating them into real-world patient outcomes, as long-term follow-up is necessary. Fifth, although novices provided positive feedback on the handmade phantom, its limitations—such as the inability to replicate the heart's kinetic motion and the lack of surrounding tissues for pericardiocentesis—may reduce the fidelity of simulated images and potentially affect performance and its transferability to real patients. This highlights the need for more advanced training models which could include high-fidelity simulations, or the use of standardized pericardiocentesis videos as an alternative to clinical observation, to improve skill retention and better prepare learners for real-world applications. Sixth, the assessment forms were developed based on a thorough literature review and expert consensus and were applied in both immediate and 3-month assessments. Although they exhibited a good reliability, we recognize that further validation of these assessment forms is necessary and recommend additional studies to confirm their utility and robustness in diverse clinical settings. Seventh, significant time and labor were required for phantom preparation. It took 30 min to cook the agar substrate and another 30 min to assemble the phantom. However, this one-hour investment in labor compensates for the high costs associated with commercial phantoms. Eighth, we gathered data on the novices’ experience regarding thoracocentesis and pericardiocentesis following training. Other potential factors such as learning from online resources or reading literature, which could have contributed to their learning, were not captured. Nevertheless, assuming uniform learning motivation among novices, the impact of these factors would likely be negligible. Last, the observed differences in learning suggest that the retraining intervals for pericardiocentesis and thoracentesis may need to differ, or that pericardiocentesis may require additional simulation time. This study focused only on the medium-term learning effects, without evaluating long-term skill retention. Extending the follow-up periods, such as 6 to 12 months and incorporating more frequent re-evaluations are essential for assessing both skill retention and the long-term efficacy of the training program. It could provide more robust data on the durability of training effects and support the development of evidence-based recommendations for the optimal frequency of refresher training.

## Conclusion

Novices exhibited superior performance in thoracocentesis but encountered a decline in skill proficiency in pericardiocentesis during the 3-month assessment following training with handmade phantoms. This disparity is likely due to the very low frequency of pericardiocentesis cases encountered by novices, along with its classification as a higher-stakes procedure. The results highlight the importance of clinical observation following training with handmade phantoms. Further investigation is needed to evaluate the long-term effects of training, skill retention, and transfer of skills to actual patient care. Additionally, research should focus on determining optimal retraining intervals for pericardiocentesis and evaluating the use of standardized pericardiocentesis videos as an alternative to clinical observation.

## Supplementary Information

Supplementary Material 1.

Supplementary Material 2.

Supplementary Material 3.

Supplementary Material 4.

Supplementary Material 5.

## Data Availability

All data analyzed during this study are included in this published article.

## Abbreviations

US: Ultrasound
EM: Emergency medicine
NTUH: National Taiwan University Hospital
PGY: Postgraduate-year
UGY: Undergraduate-year
IQR: Interquartile range
ICC: Intraclass correlation coefficient
CI: Confidence interval
