# Supplementary material for: How to enhance the novices’ learning in ultrasound-guided procedures utilizing handmade phantoms?
Source: BMC Med Educ. 2024 Dec 18;24:1444. doi: 10.1186/s12909-024-06458-z (PMC11654126; doi:10.1186/s12909-024-06458-z)
Supplement: Supplementary file 1 — Supplementary Material 1. [file 12909_2024_6458_MOESM1_ESM.docx]

Supplementary Table 1. The details of the cost of the hand-made phantoms.

| Item | Thoracocentesis | Pericardiocentesis |
| --- | --- | --- |
| Agar substrate |  |  |
| Agar powder, 0.4 USD/10g | 4 | 4 |
| Dark blue food coloring additives, 1 USD/10cc | 1 | 1 |
| Super glue | 0.3 | 0.3 |
| Container | 5 | 5 |
| Ziploc bag, 2 USD/bag | 2 | - |
| Tongue depressor, 0.5 USD/10 pieces | 0.25 | - |
| Food coloring additives. 1 USD/10cc | 0.2 (yellow) | 0.4 (yellow, red) |
| 6-inch balloon, 3 USD/balloon | - | 3 |
| 3-inch balloon, 1 USD/balloon |  | 1 |
| Total cost, USD | 12.75 | 14.7 |
