# Supplementary material for: How to enhance the novices’ learning in ultrasound-guided procedures utilizing handmade phantoms?
Source: BMC Med Educ. 2024 Dec 18;24:1444. doi: 10.1186/s12909-024-06458-z (PMC11654126; doi:10.1186/s12909-024-06458-z)
Supplement: Supplementary file 3 — Supplementary Material 3. [file 12909_2024_6458_MOESM3_ESM.docx]

Supplementary Table 3. The assessment form for pericardiocentesis.

| Item | 1 | 2 | 3 | 4 | 5 |
| --- | --- | --- | --- | --- | --- |
| Visualization of heart, pericardium, and effusion | No recognizable structures | Minimally recognizable structures but insufficient for diagnosis | Recognizable structures | Recognizable structures and all structures imaged well | all structures imaged with excellent image quality |
| Visualization of needle | Needle tip not seen during entering and difficult to locate after entry |  | Needle tip not seen during entering but easily and quickly located after entry |  | Tip clearly seen during entering |
| Puncture and fluid aspiration | No fluid is aspirated |  | Fluid is aspirated |  | Fluid is aspirated smoothly |
| Needle steadiness during aspiration | Many unnecessary movements; the needle touched the heart |  | Some unnecessary needle movements |  | Minimal needle movement during aspiration |
| Global scores | Unacceptable performance with multiple major deficiencies across all assessed items | Unacceptable performance; some major inadequacies across all assessed items | Acceptable performance; minor inadequacies across all assessed items | Acceptable performance that meets the requirements across all assessed items | Expert performance |
