# Supplementary material for: How to enhance the novices’ learning in ultrasound-guided procedures utilizing handmade phantoms?
Source: BMC Med Educ. 2024 Dec 18;24:1444. doi: 10.1186/s12909-024-06458-z (PMC11654126; doi:10.1186/s12909-024-06458-z)
Supplement: Supplementary file 5 — Supplementary Material 5. [file 12909_2024_6458_MOESM5_ESM.docx]

Supplementary Table 5. The interrater reliability for pericardiocentesis assessment.

| Assessment | Score^*^ | Intraclass correlation coefficient (95% CI^†^) |
| --- | --- | --- |
| Visualization of heart, pericardium, and effusion | 3 (3-5) | 0.92 (0.86-0.95) |
| Visualization of needle | 3 (3-5) | 0.97 (0.94-0.98) |
| Puncture and fluid aspiration | 5 (3-5) | 0.96 (0.93-0.98) |
| Needle steadiness during aspiration | 5 (3-5) | 1 |
| Total score | 16.5 (14-18) | 0.99 (0.98-0.99) |
| Global score | 3 (3-4) | 0.94 (0.90-0.97) |

^*^presented with median and interquartile ranges.

^†^CI=confidence interval.
